# Novel Flow Cytometry Method Detecting Complement C1q Bound to Blood Type A/B IgG Antibody for Preventing Severe Antibody-Mediated Rejection in ABO-Incompatible Kidney Transplantation

**DOI:** 10.3390/antib13030062

**Published:** 2024-08-01

**Authors:** Tsutomu Ishizuka, Kazuhiro Iwadoh, Hiroshi Kataoka, Junichi Hoshino, Kosaku Nitta, Hideki Ishida

**Affiliations:** 1 Department of Transplant Immunology, Tokyo Women’s Medical University, Tokyo 162-8666, Japan; ishizuka.tsutomu@twmu.ac.jp; 2 Department of Transplant Surgery, Mita Hospital, International University of Health and Welfare, Tokyo 108-8329, Japan; 3 Department of Nephrology, Tokyo Women’s Medical University, Tokyo 162-8666, Japan; 4 Department of Urology, Tokyo Women’s Medical University, Tokyo 162-8666, Japan

**Keywords:** blood type A/B antibody, complement-binding blood type antibody, ABO blood type-incompatible, kidney transplantation, hyperacute rejection

## Abstract

We aimed to develop a novel method for measuring the complement-binding ability of anti-blood type antibodies (ab-Abs), the flow cytometry method for the complement C1q test (FCM-C1q) for detecting antibody-mediated rejection (AMR) caused by ab-Abs in ABO-incompatible kidney transplantation (ABOI-KTx). FCM-C1q distribution was surveyed in 44 healthy participants and 43 dialysis patients (Cohort A). The relationship between AMR and FCM-C1q levels was examined along with ab-Ab titers by the flow cytometry method for the IgG test (FCM-IgG) in 62 ABOI-KTx patients (Cohort B). FCM-IgG and C1q levels were significantly higher in type O participants than in A/B participants in Cohort A. There were minimal differences in the distribution of FCM-IgG and C1q between dialysis and healthy participants. Sixteen cases were suspected of acute rejections (ARs) in Cohort B, of whom nine had AR clinically. One patient with severe AMR was highly suspected of hyperacute rejection along with another patient with severe AMR. Their postoperative FCM-C1q and FCM-IgG levels were elevated. Another two patients showed high FCM-IgG and C1q levels before KTx, and these levels remained low after KTx with no or mild rejection. In conclusion, our results suggest that a high positivity rate for FCM-C1q may predict moderate to severe AMR caused by ab-Abs and poor prognosis in ABOI-KTx.

## 1. Introduction

ABO blood type-incompatible kidney transplantation (ABOI-KTx) necessitates splenectomy in conjunction with plasmapheresis (PPx) to mitigate antibody-mediated acute rejection caused by anti-blood type antibodies (ab-Abs) immediately post-surgery [1,2,3,4]. However, considerable advancements were made in 2002 when the chimeric anti-CD20 monoclonal antibody rituximab (RXM) (Genentech, San Francisco, CA, USA) was introduced to prevent anti-blood type antibody-mediated rejection (abAMR) [5]. This development has enabled ABOI-KTx procedures to be performed without splenectomy [6]. Additionally, anti-blood type A/B antibodies, the primary culprits of abAMR, can be removed using antibody removal therapies such as double-filtration PPx or plasma exchange. These interventions have significantly improved the graft survival rate for ABOI-KTx [7,8]. Although these preconditioning procedures do not achieve complete antibody depletion, post-transplantation renal conditions are satisfactory in most instances. However, we speculate that there have been sporadic occurrences of hyperacute antibody-mediated rejection (HAR), the immunological tests for which remain largely unexplored, resulting in the underreporting of this condition [9,10].

Our previous study highlighted that donor-specific anti-HLA-IgG antibodies (DSAs), when combined with complement-binding DSA, have a strong correlation with humoral rejection in patients in whom there is a mismatch in HLA antigens between the donor and the recipient [5]. As such, we hypothesized that complement-binding anti-A/B antibodies may be strongly linked to abAMR in ABOI-KTx, given that they are inherently part of the immune system. To validate this hypothesis, we introduced a novel method in our pilot study that used human complement C1q, the initial component of the complement system, and assessed its efficacy in this preliminary pilot study reviewing the clinical consequences of ABOI-KTx. This method was the flow cytometry method for the complement C1q test, which we named FCM-C1q. The conventional method for measuring antibody titers of anti-A/B blood type IgG antibodies with the flow cytometry method was named FCM-IgG.

## 2. Materials and Methods

### 2.1. Study Population

This study examined two groups of people, Cohorts A and B. Cohort A consisted of 44 healthy individuals including 30 renal donors and 43 patients on dialysis scheduled for ABOI-KTx to observe how the results of two specific tests (FCM-IgG and FCM-C1q) varied between them. Cohort B comprised 62 (29.1%) out of 213 kidney transplant recipients who underwent transplantation at the Department of Surgery III, Tokyo Women’s Medical University, between January 2014 and December 2015. This group was evaluated to verify if the FCM-C1q test could predict or confirm antibody-mediated rejection (AMR) due to mismatched blood types in a clinical setting. This study had no specific exclusion criteria for the participants.

#### 2.1.1. Flow Cytometry Method for IgG Test

**Figure 1:**
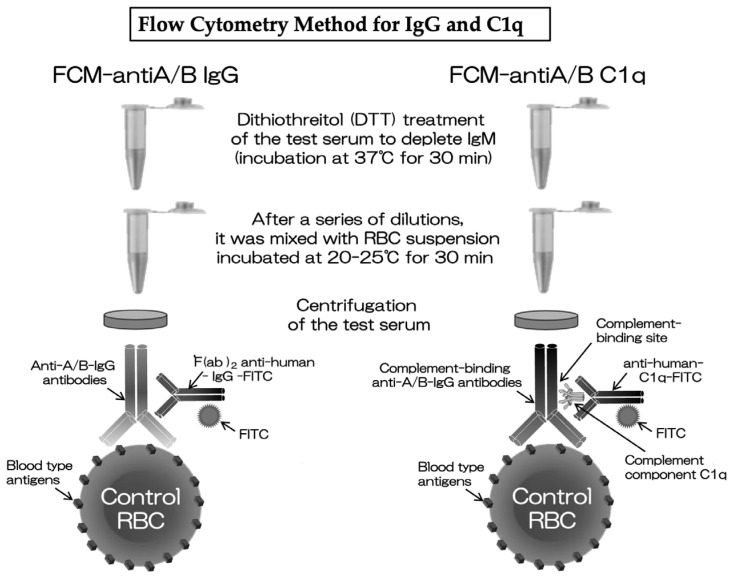
A schematic diagram of the FCM-IgG and FCM-C1q assay methods.

Anti-A/B-IgM antibody depletion was performed by treating the test serum with 50 mM dithiothreitol (DTT; FUJIFILM Wako Pure Chemical Corporation Inc., Osaka, Japan) at a 1:9 ratio at 37 °C for 30 min. Then, the DTT-treated test serum was diluted with a dilution solution (phosphate-buffered saline [pH 7.4] Gibco, Waltham, MA, USA) in a series to make several samples diluted stepwise to 2-fold, 4-fold, 8-fold, and higher (Supplementary Figure S1). A total of 50 μL of diluted serum was taken from each sample, and 50 μL of red blood cell suspension (RBC-S) (DiaCell ABO A1-B, DiaMed AG, Cressier, FR, Switzerland) was mixed. Next, the negative control was made by mixing 50 μL of AB-type human serum commercially available with 50 μL of RBC-S, and the positive control was made by mixing 50 μL of the positive anti-A/B IgG antibody serum from blood type O with 50 μL of RBC-S. The series of diluted sera, negative control, and positive control were incubated at room temperature (20–25 °C) for 30 min. The reacted erythrocytes were washed three times while spun down at 17,922× *g* (14,680 rpm) for 1 min. Afterward, 50 μL of fluorescein isothiocyanate (FITC)-conjugated anti-human IgG (Jackson Immunoresearch Laboratories, West Grove, PA, USA) was diluted 300 times, added to each sample, and incubated in the dark at room temperature for 20 min. The reacted red blood cells were washed once more while spun down. Finally, FCM-IgG levels were measured using the BD FACSCanto II flow cytometer (Becton Dickinson and Company, San Jose, CA, USA). The FCM-IgG levels were determined by the final positive titer in the dilution series (2*^n^*) [11]. The FCM-IgG assay method is illustrated in a schematic diagram in Figure 1.

#### 2.1.2. Flow Cytometry Method for Complement C1q Test

The test serum was heated at 56 °C for 30 min to inactivate the complement in the serum. The test serum treated with DTT, similar to FCM-IgG, was incubated at 37 °C for 30 min to remove anti-A/B-IgM antibodies [12,13]. Next, 50 μL of the test serum was mixed with 50 μL of RBC-s (DiaCell ABO A1-B, DiaMed AG, Cressier, FR, Switzerland). Then, a negative control was created by mixing 50 μL of commercially available human serum type AB with 50 μL of RBC-s, and a positive control was created by mixing 50 μL of the positive anti-A/B-C1q serum from type O blood with 50 μL of RBC-s. The test serum, negative control, and positive control were incubated at room temperature (20–25 °C) for 30 min. After being washed three times while spun down at 17,922× *g*, 5 μL of the C1q component (Sigma-Aldrich, St. Louis, MO, USA) was added and incubated at room temperature for 20 min. Finally, 50 μL of FITC-labeled anti-human C1q (Abcam, Cambridge, MA, USA), diluted 200 times, was added and incubated in the dark at room temperature for 20 min. The reacted red blood cells were washed once under the same spin-down conditions, and the FCM-C1q levels were measured using the BD FACSCanto II flow cytometer, with results determined on the basis of the positivity rate (percentage) as stated below. The FCM-C1q assay method is also shown in Figure 1.

The method for counting positive cells in FCM-C1q is as follows. First, a histogram of red blood cell counts in the test serum according to the mean fluorescence intensity was generated using a commercial AB-type serum as the negative control (Figure S2). In the flow cytometer, BD FACSCanto II (Becton Dickinson and Company), the red blood cells that had reacted with the AB-type serum were gated. The flow cytometer was set to automatically stop once 10,000 red blood cells within the gate had been captured. Then, a cut off line was set to include more the 99.0% of the negative control cells, dividing the graph space into the positive and negative regions. The positivity percentage of the patients’ red blood cells was determined by the number of cells in the positive region divided by the total number of the red blood cells analyzed, which was calculated using FlowJo version 10.0 (BD, San Diego, CA, USA).

FCM-IgG assay method: The test serum is treated with DTT to remove IgM antibodies and then diluted in a series such as 2-fold, 4-fold, 8-fold, and higher (Supplementary Figure S1). The red blood cell suspension is added to each diluted sample and incubated for 30 min. The same procedure is applied to both AB-type human serum as a negative control and positive A/B IgG antibody serum as a positive control. After being washed three times with centrifugation, the reacted red blood cells are mixed with anti-human-IgG-FITC and incubated for 20 min. After being washed once, the fluorescence intensity of FITC is measured by flow cytometry at the final positive titer in serial dilutions of the test serum.

FCM-C1q assay method: The test serum is heated to inactivate the complement and treated with DTT to remove IgM antibodies. The test serum is mixed with red blood cell suspension and incubated at room temperature for 30 min. The same procedure is applied to both AB-type human serum as a negative control and positive A/B IgG antibody serum as a positive control. After being washed three times with centrifugation, the reacted red cells are mixed with C1q complement and incubated for 20 min. Next, without a wash, FITC-labeled anti-human C1q is added and incubated for another 20 min. Following a single wash, the intensity of FITC is measured using flow cytometry.

Molecular mechanism of FCM-C1q: Patients’ complement-binding anti-A/B IgG antibodies bind to commercially available red blood cells, and human complement component C1q (hcc-C1q) binds to their complement-binding site (C1q binding site). Then, anti-human-C1q-FITC, which recognizes hcc-C1q, is bound to hcc-C1q, and FCM-C1q is measured by the fluorescence intensity of FITC, the fluorescent labeling reagent anti-human-C1q-FITC possesses.

FCM-IgG, flow cytometry method for the IgG test; FCM-C1q, flow cytometry method for the complement C1q test.

### 2.2. Immunological Regimen for ABOI-KTx

The immunological regimen for ABOI-KTx is depicted in Supplementary Figure S3. Recipients commenced oral administration of 1 mg tacrolimus (Tac) and 250 mg mycophenolate mofetil (MMF) 30 days prior to hospitalization. Subsequently, the Tac dosage was increased to achieve a trough concentration between 10 ng/mL and 15 ng/mL by the 5th postoperative day (POD). The MMF dosage was also increased to 2000 mg/day on the same day. These dosages were maintained throughout the hospital stay. As part of the induction therapy, 100 mg RXM was administered on POD -5, and 40 mg basiliximab (BXM) was administered on POD 0 and POD 4. RXM was not used in patients whose hepatitis B virus (HBV)-DNA status was positive or who had undergone splenectomy. To remove ab-Abs, PPx with double-filtration plasmapheresis (DFPP) or plasma exchange was conducted on POD -5, POD -3, and POD -1. Methylprednisolone (MP) administration commenced daily from POD 0, with the dosage gradually reduced from 500 mg to 4 mg by POD 14. MP administration was discontinued in patients with a low risk of rejection. An antiplatelet drug was administered after hospitalization to all patients who was scheduled to receive ABOI-KTx to prevent thrombotic microangiopathy (TMA).

### 2.3. Anti-A/B Antibody Measurement in Dialysis Patients and Healthy Participants

Flow cytometry for IgG and C1q was performed as stated above in the first cohort. The amounts of blood type antibodies were then compared between type O and type A/B participants among healthy participants and among dialysis patients. They were also compared between dialysis patients and healthy participants according to blood type.

### 2.4. Anti-A/B Antibody Measurement in ABOI-KTx before and after Transplantation

The patient characteristics, including age, sex, spousal donor, dialysis period, HLA matching, FCM-IgG/-C1q pre- and post-transplantation and induction therapy (e.g., BXM, RXM (100 mg/body), and anti-thymoglobulin (ATG)), were analyzed in Cohort B. HLA matching indicated the number of different amino acids in the estimated amino acid sequences of HLA molecules between the donor and the recipient. Background patient characteristics during hospitalization for transplantation were then compared between the rejection group (RG) and the non-rejection group (NRG), with the RG involving patients suspected of rejection.

When the serum creatinine (s-Cr) level after KTx increased by more than 20% from its nadir with no other obvious indication besides rejection, it was clinically considered at least a suspected acute rejection (sAR). Owing to the administration of antiplatelet drugs in ABOI-KTx according to the protocol, performing renal biopsy was mostly difficult when rejection was suspected. Therefore, determining whether the rejection was T-cell-mediated rejection or AMR and whether the AMR was caused by anti-HLA antibodies (ah-Abs) or ab-Abs had to be judged on the basis of the participants’ clinical course. Furthermore, steroid pulse therapy was performed as a treatment that also served as a diagnostic tool for estimating the nature of rejection such as a mild T-cell-mediated rejection, which could often occur in the early stage after KTx. If the condition was resolved only with steroids, it was classified as sAR. If impaired renal function was not recovered by steroid pulse therapy, it was classified as AR, and additional therapies including plasma exchange or gamma globulin or ATG administration were performed according to the severity of renal dysfunction. Even if these criteria for rejection were not met, a diagnosis of rejection was made if rejection could not be ruled out.

The preoperative FCM values were primarily from POD -2. However, if a serum sample from that day was not available, test results from other days were used as substitutes. Postoperative FCM values were primarily from POD 4. However, if a sample from that day was not available, test results from a nearby day were used. Additionally, in cases where abAMR occurred, the test results from the day when FCM levels were at their peak were used; however, due to the varying day of rejection, there was inconsistency in test days. In some cases, all values of FCM IgA/C1q performed before and after surgery are illustrated in Supplementary Figures S1–S13, as discussed in the Section 3 and Section 4.

Recipients who developed rejection during hospitalization were surveyed, with a focus on their immunological test results, induction, types of rejection, treatment, and prognosis. For those whose FCM-C1q exceeded 10% before or after transplantation, the detailed clinical course, including FCM-IgG, FCM-C1q, Cr level, and treatments for rejection, was examined. Although it was desirable to set the threshold of FCM-C1q as close to 0% as possible, it was determined empirically on the basis of our clinical experiences using FCM-C1q on ABOI-KTx, while 5–10% of FCM-C1q was considered the judgment-pending zone.

### 2.5. Statistical Analysis

Continuous variables are represented as mean ± standard deviation (minimum value–maximum value). Discrete values were tested using the chi-square test or Fisher’s exact test, and continuous values were compared using the Mann–Whitney U test or the Kruskal–Wallis test with Mathematica 13.0 (Wolfram Research Inc., Champaign, IL, USA) and JMP Pro11.2.0. A *p*-value of <5% was considered significant.

## 3. Results

### 3.1. Demographics and Blood Groups of Cohort A

There were no significant differences in age or sex across blood types in healthy participants (age, *p* = 0.233; sex, *p* = 0.291) or in dialysis patients (age, *p* = 0.0524; sex, *p* = 0.301). The characteristics of Cohort A are shown in Table 1.

#### Comparison of Anti-A/B Antibodies between Healthy Individuals and Dialysis Patients

**Figure 2:**
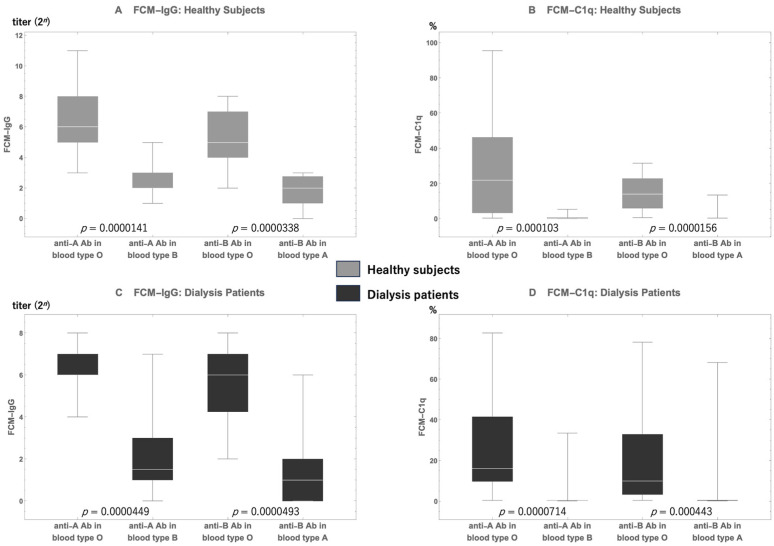
A comparison of anti-A/B antibody titers (**A**,**C**) and their C1q-positive rates (**B**,**D**) between blood type O participants and blood type A/B participants. In both healthy individuals and dialysis patients with blood type O, both the FCM-IgG antibody titers and the FCM-C1q positivity rates are significantly higher than those in blood type A and B participants. FCM-IgG, flow cytometry method for the IgG test; FCM-C1q, flow cytometry method for the complement C1q test.

Figure 2 shows the comparisons between blood type anti-A/B antibodies in type O participants and those in type A/B participants in each group. Figure 2A,B present the data of the healthy participants, whereas Figure 2C,D show the data of the patients on dialysis. Both FCM-IgG antibody titers (Figure 2A,C) and FCM-C1q-positive rates (Figure 2B,D) are provided, highlighting the differences in antibody levels across the blood types.

FCM-IgG anti-A/B antibody titers were significantly higher in type O participants than in type B/A participants, both in healthy participants and dialysis patients. These tendencies of distributions of FCM-IgG antibodies were also similar to those of FCM-C1q-positive rates. FCM-C1q-positive rates in type O participants were significantly higher than those in blood type B/A participants in both groups.

**Figure 3:**
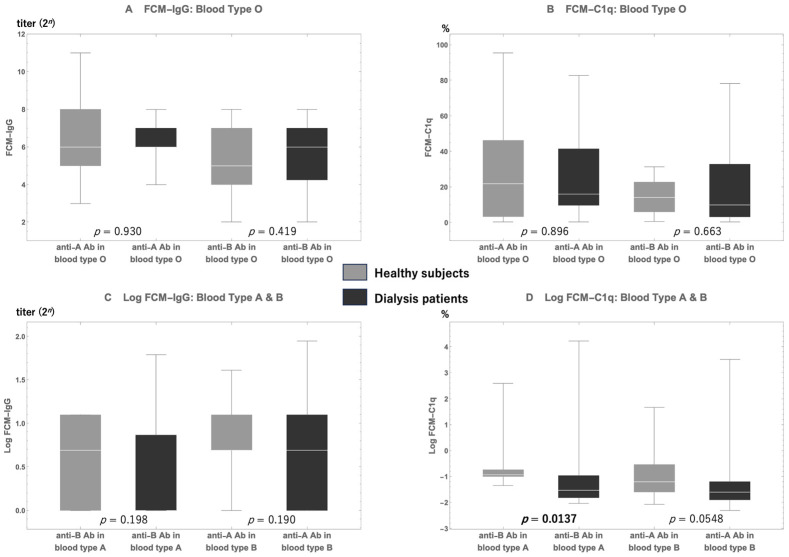
A comparison of anti-A/B antibody titers (**A**,**C**) and their C1q-positive rates (**B**,**D**) between healthy individuals and dialysis patients. For both anti-A and anti-B antibody titers of FCM-IgG and FCM-C1q, there are no significant differences between healthy participants and dialysis patients, except for the FCM-C1q-positive rates of anti-B antibody titers (**D**). FCM-IgG, flow cytometry method for the IgG test; FCM-C1q, flow cytometry method for the complement C1q test.

When the amounts of the same blood type antibodies in the same blood type group were compared between healthy participants and dialysis patients (Figure 3), there were no significant between-group differences, except for the FCM-C1q level of anti-A antibody in dialysis patients with blood type B that was significantly lower than that in dialysis patients (*p* = 0.0137; Figure 3D).

### 3.2. Demographics of Cohort B

The average donor age was 60.8 ± 9.1 years (range, 40–79 years), with 20 males and 42 females. A total of 32 donors (51.6%) were spousal. The average recipient age was 48.9 ± 13.9 years (range, 17–77 years), with 42 males and 20 females. The mean dialysis period was 4.93 ± 7.07 years (range, 0.01–29.6 years). The average mismatch count of amino acids in the estimated sequence of HLA A, B, and DR proteins between the donor and the recipient was 35 ± 17.7 (range, 0–76). As induction therapy, BXM, RXM, and ATG were used in 61, 59, and 10 recipients, respectively.

#### 3.2.1. Comparison of Patient Characteristics between Rejection and Non-Rejection Groups

A total of 16 rejections, including nine AR and seven sAR, were observed during hospitalization after KTx in Cohort B. Table 2 shows the comparison of background characteristics between the RG and NRG. No significant difference was found between them. The average FCM-IgG before KTx in the RG and NRG were 10.0 ± 20.21 and 5.48 ± 7.66, respectively (*p* = 0.647), and those after KTx were 7.81 ± 16.85 and 2.67 ± 3.60, respectively (*p* = 0.520). The average FCM-C1q before KTx in the RG and NRG were 7.81 ± 16.85 and 2.67 ± 3.60, respectively (*p* = 0.520), and those after KTx were 4.49 ± 10.85 and 0.91 ± 0.53, respectively (*p* = 0.748). Other immunological factors, including crossmatch test results and induction therapies, were comparable between them. Preformed DSA was found in two recipients (12.5%) in the RG and in four patients (8.7%) in the NRG (*p* = 0.643).

A comparison of patient background between 9 AR cases, 7 sAR cases, and 46 no-rejection cases was also performed, as shown in Supplementary Table S1. No clinically meaningful difference was found between these cases.

#### 3.2.2. Immunological Test Results and Clinical Courses of Recipients in RG

**Figure 4:**
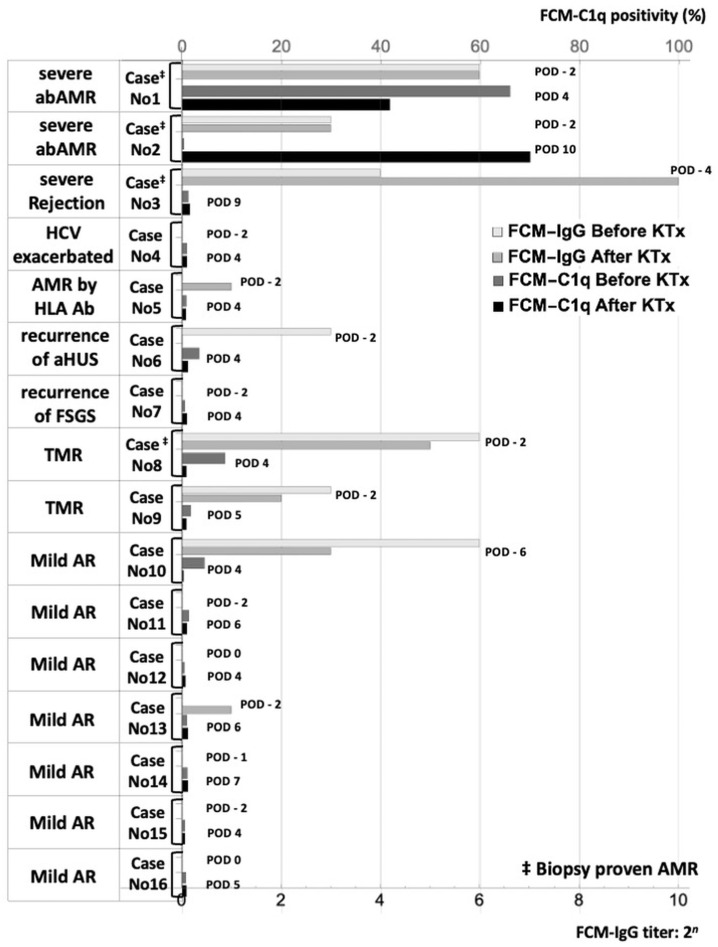
A bar chart plot of FCM-IgG and FCM-C1q values for the 16 cases who developed rejection.

Supplementary Table S2 demonstrates the immunological test results and clinical courses of all cases, including the 16 recipients who developed rejection. Figure 4 depicts the preoperative and postoperative FCM-IgG and FCM-C1q values of these 16 cases in a bar chart plot.

Two recipients developed biopsy-confirmed antibody-mediated acute rejection. Preoperative immunological tests concerning HLA were all negative in these patients. One patient (Case No. 1) was a 60-year-old female whose donor was her husband. Her preoperative FCX-IgG titer was 64 times, and the FCX-C1q-positive rate was as high as 66.0%. Intraoperatively, the color of the transplanted kidney darkened, and the diastolic phase disappeared on a Doppler echo image. Suspecting rejection, it was removed for reperfusion, but the reperfusion fluid did not flow at all due to the formation of thrombi in the arteries, leading to the decision of graftectomy. The removed kidney was reddish-purple (Supplementary Figure S4). The pathological diagnosis was HAR. The gross and microscopic images of the graft are shown in Supplementary Figure S5. Diffuse glomerular TMA was found. Her FCM-IgG titer was still 64 times, and her FCM-C1q-positive rate was still high at 41.7% on POD 4. The detailed FCM test results are supplied in Supplementary Figure S6.

The other patient (Case No. 2) was a 65-year-old male whose donor was his wife. His preoperative FCX-IgG and FCM-C1q were as low as one time and 0.7%, respectively. His FCM-C1q started to increase on POD 8 along with his serum creatinine (s-Cr) level (Supplementary Figure S7). On POD 10, his FCM-C1q was as high as 70% with s-Cr being 3.11 mg/dL; meanwhile, his FCM-IgG titers were stable at around 2^3^. The pathological diagnosis of his graft was acute and active AMR. The treatment for rejection started with steroid pulse therapy, followed by the administration of ATG and gabexate mesylate, a drug for disseminated intravascular coagulation. On the basis of the renal biopsy result on POD 21, eight sessions of consecutive plasma exchanges were conducted, resulting in FCM-C1q decreasing to 0.332% on POD 30 and a gradual decrease in s-Cr to 2.35 mg/dL at discharge.

This figure contrasts the preoperative and the postoperative levels of FCM-IgG and FCM-C1q along with the type and severity of clinical AR in each case. The unit of the horizontal axis was the titer for FCM-IgG (bottom horizontal line) and the percentage of C1q-positive anti-A/B IgG antibodies for FCM-C1q (top horizontal line). The test date for the FCM method is displayed as the POD to the right of the test values. In Case No. 1, the FCM-C1q level was highly elevated before and after KTx leading to HAR. In Case 2, although the preoperative FCM-C1q level was decreased, the postoperative FCM-C1q level rebounded from POD 8 to reach 70%, accompanied by severe abAMR. In Case No. 3, the FCM-C1q level was not measured during the period of rejection. All the other cases did not show an elevation in the FCM-C1q level after ABOI-KTx without moderate or severe abAMR. FCM-IgG, flow cytometry method for the IgG test; FCM-C1q, flow cytometry method for the complement C1q test; KTx, kidney transplantation; No., number; abAMR, antibody-mediated rejection against anti-blood type antibodies; HCV, hepatitis C virus infection; HLA ab, anti-HLA antibodies; aHUS, atypical hemolytic uremic syndrome; FSGS, focal segmental glomerulosclerosis; TMR, T-cell-mediated rejection; AR, acute rejection.

Among the other seven AR patients, Case No. 3, who underwent a second KTx, exhibited an increase in s-Cr starting from POD 7, reaching to as high as 4.70 mg/dL by POD 10. This occurred alongside an increase in the FCM-IgG levels, which reached 1024 times higher by POD 9. However, the FCM-C1q level was 1.44% on POD 9 (Supplementary Figure S8). Considering his ah-Ab was negative after KTx, it is possible that abAMR occurred. In that case, the reason the rise in FCM-C1q was not observed could be attributed to FCM-C1q not being measured between POD 4 and POD 8, as well as the administration of plasmapheresis and gamma globulin during that period. The low C1q level on POD 9 is presumed to be the result of absorption of the antibody by the renal graft. The pathological findings from a renal biopsy on POD 34 indicated histological appearances consistent with AMR that had already been treated. Case No. 4 was not administered RXM owing to a positive HBV-DNA status. Therefore, strictly speaking, he represented a case that deviated from the standard protocol for ABOI-KTx. He also had a history of brainstem hemorrhage, triple-vessel coronary artery disease, and chronic obstructive pulmonary disease. His KTx was conducted at his strong request, and he was fully aware of the risks. Immediately after surgery, kidney function was poor, with creatinine levels decreasing to approximately 5 mg/dL, resulting in an acute exacerbation of HCV hepatitis and finally leading to liver failure. Because his FCM-C1q levels remained at less than 3.6% throughout the course, we estimated that his clinical AR was not facilitated by abAMR.

For Case No. 5, the CDC and FXCM of B-cells and PRA class 2 were all positive with preformed DSA preoperatively, indicating a high likelihood of AMR against ah-Abs. For Case No. 6, the primary disease was atypical hemolytic uremic syndrome (aHUS), and her clinical AR was probably due to the recurrence of aHUS. For Case No. 7, his primary disease was focal segmental glomerulosclerosis and his renal function manifested very slowly from immediately after surgery, indicating that his clinical AR was due to the recurrence of his primary disease. For Case No. 8, his FCM-IgG and C1q levels before KTx were high at 64 times and 8.6%, respectively, on POD -2. After KTx, his FCM-IgG and C1q levels were reduced to 32 times and 0.7%, respectively. The pathological finding on POD 20 was no apparent change, suggesting that his AR was reversible T-cell-mediated rejection (TMR). For Case No. 9, he showed a slow decrease in Cr, with a Cr of 3.22 mg/dL and low FCM-IgG levels with anti-A antibody at 4 times and anti-B antibody at 16 times on POD7. Therefore, he was clinically diagnosed as TMR and treated with ATG and gamma globulin. His pathological finding on POD 104 was acute TMR.

In seven patients with sAR, sAR was alleviated only through steroid pulse therapy, suggesting that their sAR was steroid-reactive TMR or another type of mild rejection. Their FCM-IgG and FCM-C1q levels remained low throughout the clinical course, except for Case No. 10. In this patient, FCM-IgG and FCM-C1q levels were 64 times and 14.20%, respectively, on POD -6, and they decreased to two times and 1.76%, respectively, just before KTx with preconditioning treatments. Postoperatively, her FCM-C1q levels remained as low as <1% only with steroid-reactive AR, while FCM-IgG levels increased to 32 times by POD 11 without any change in her renal function. These findings are depicted in Supplementary Figure S9.

Among the 46 patients without rejection during hospitalization, Case Nos. 17 and 18 exhibited elevated FCM-IgG and FCM-C1q levels before surgery (Supplementary Figures S10 and S11). For Case No. 17, FCM-IgG and FCM-C1q levels were 64 times and 88.9% higher, respectively, on POD -6. After KTx, these levels decreased to eight times and 0.4%, respectively, by POD 4, with stable renal function maintained. In Case No. 18, FCM-IgG and FCM-C1q levels were 32 times and 19.0%, respectively, only before KTx, and they decreased to 8 times and 1.4%, respectively, by POD 6, with an s-Cr level of 1.1 mg/dL. Other patients with sAR did not show elevated levels of either FCM-IgG or FCM-C1q after KTx.

## 4. Discussion

In this study, increased FCM-C1q levels, along with elevated FCM-IgG titers after ABOI-KTx, were associated with moderate to severe abAMR, particularly in Case Nos. 1 and 2. Although severe abAMR after ABOI-KTx is rare nowadays with standard preconditioning using RXM and PPx, we encountered a few patients in which such as abAMR was highly suspected. Further, high FCM-IgG levels did not necessarily lead to abAMR or only led to mild abAMR when FCM-C1q levels were low, as demonstrated by Case Nos. 10 and 17. Therefore, we propose that continuous assay of pre- and postoperative levels of FCM-C1q, along with those of FCM-IgG, may be useful for predicting and monitoring moderate to severe abAMR in ABOI-KTx during the perioperative period, which could potentially be fatal.

Alexandre et al. [14,15] reported that thorough preoperative anti-blood group antibody removal coupled with splenectomy facilitated a successful ABO-incompatible kidney transplant. In the early days of ABOI-KTx, he reported nine such patients, and three of these patients who did not undergo splenectomy developed irreversible rejection within a week after KTx. This rejection, which probably would have been severe abAMR, necessitated graftectomy [6]. This shows that severe abAMR may probably occur in ABOI-KTx if appropriate preconditioning is not performed.

In Japan, ABO blood group-incompatible live-donor kidney transplantation was introduced in 1989 with the aim of widening the kidney donor pool. The removal of anti-A/B antibodies along with RXM induction was associated with higher kidney transplant survival rates than conventional standard procedures using splenectomy [14,15,16]. Current epidemiological evidence suggests that total ab-Ab removal is not necessary [17]. However, the exact number of antibodies needed for successful grafting remains unclear, with practices largely based on clinical experience [18]. Several methods, including FCM-IgG, gel column centrifugation (MTS; DiaMed-ID Micro Typing System, DiaMed AG, Cressier FR, Switzerland), bead column centrifugation (CAT; BioVue Column Agglutination Technology, Ortho-Clinical-Diagnostics, Tokyo, Japan), and flow cytometry, have been used to measure blood type anti-A/B antibodies. Notably, these techniques detect all IgG subclasses (IgG 1–4), and their foundational concepts remain largely unchanged.

We hypothesized that compared with indiscriminately removing as many antibodies as possible, removing anti-A/B IgG antibodies capable of binding to the complement before surgery was more critical, regardless of their ability to bind to the complement, for achieving successful ABOI-KTx [19]. There are reports of patients with accelerated AMR in ABOI-KTx who were rescued from graft loss by using eculizumab, which inhibits the complement cascade system [20,21,22]. Given that FCM-C1q is a novel method, we initially examined the distributions of FCM-C1q-positive rates among dialysis patients and healthy participants and compared the differences in FCM-C1q levels according to blood type in both groups. In Cohort A, there was a significant difference between anti-A/B IgG antibody titers in type O, type A, and type B participants, among both healthy participants and dialysis patients (Figure 2). Further, we found that the FCM-C1q positivity rate was significantly higher in type O patients than in type A/B patients. Anti-A/B IgG antibody titers across types O, A, and B in dialysis patients closely resembled those of their healthy counterparts (Figure 2). The distributions of FCM-C1q-positive rates were also similar across all blood types between healthy participants and dialysis patients, except for the C1q level for anti-B antibody in type A, the rate of which was significantly higher in healthy participants than in dialysis patients.

Some studies have suggested that the effect of sex-related differences on anti-A/B IgG antibodies is more pronounced in female patients than in male patients [23,24]. Thus, women may have more opportunities to become sensitized to blood group antigens than men, especially due to experiences such as pregnancy. Yabe et al. [25] noted a decline in anti-A/B antibody titers in the Japanese population in recent years. Our findings corroborate this trend, highlighting reduced anti-A/B antibody titers in type A and type B participants. However, anti-A/B antibodies in type O participants demonstrated sustained high titers, with a correspondingly elevated FCM-C1q positivity rate and high levels of complement-fixing antibodies. Ishida et al. [26] observed that in the context of ABOI-KTx desensitization, type O dialysis patients exhibited a distinct post-desensitization rebound frequency, suggesting stronger immune sensitivity than type A and B dialysis patients. Our hypothesis attributes this observation to the presence of complement-fixing anti-A/B IgG antibodies in the type O blood group.

We first encountered a rare patient that was highly suggestive of HAR in 2014. We had conducted more than 300 ABOI-KTx since 1990. Following the introduction of BXM and RXM, ABOI-KTx had become a quite stable procedure without HAR. Case No. 1 was our first patient with HAR. Although we aimed to reduce the FCM-IgG titers to less than 32 times in our protocol, there have been various reports regarding preoperative antibody removal. We had also experienced some patients who proceeded to KTx without antibody removal due to the initial low titers and patients who achieved successful ABOI-KTx with FCM-IgG titers at 64 times. As for Case No. 1, DFPP was conducted once on POD -2, and MMF was switched to mizoribine due to severe gastrointestinal side effects. After KTx failure that was strongly suggestive of HAR, we suspected a rejection due to HAR caused by ab-Abs because her immunological tests concerning HLA were all negative. This resulted in the development of a novel immunological test of FCM-C1q for detecting the complement-binding ability of ab-Abs. The mechanism of FCM-C1q from the molecular viewpoint is depicted in Figure 1 along with a brief description of its assay method.

In the development of the FCM-C1q method, we have speculated that the basic mechanism for activating antibodies with C1q is similar between ah-Ab and ab-Ab; moreover, moderate-to-severe humoral rejection caused by ab-Abs is mainly associated with IgG, similar to the humoral rejection observed with ah-Abs. We report a rare case in which complement-dependent cytotoxicity positivity resulted from anti-HLA IgM antibodies against donor-specific antigens [27]. In this case, despite the presence of high levels of anti-HLA IgM antibodies, the patient did not experience substantial rejection, suggesting a different immunological impact of IgM antibodies compared with that of IgG. Pathological findings indicated only mild AMR. Additionally, although pentameric IgM antibodies can efficiently activate the complement system in its early stage, they do not induce antibody-dependent cell cytotoxicity, unlike IgG antibodies. Moreover, one study reported that anti-HLA IgM antibodies protect organs from injuries induced by IgG antibodies [28]. Therefore, DTT treatment was performed at the beginning of the FCM-C1q method to accurately quantify IgG antibodies bound to C1q.

In Cohort B, which included Case No. 1, we examined the relationship between FCM-C1q and abAMR in detail. Case Nos. 1 and 2 developed AMR and exhibited elevated levels of FCM-C1q with high FCM-IgG titers, suggesting that the presence of high FCM-C1q levels, regardless of their preoperative status, signified the occurrence of abAMR. This indicates that an increase in complement-binding ab-Abs postoperatively might serve as a biomarker for abAMR. To reinforce this hypothesis, historical records were searched for ABOI-KTx patients with preserved serum in whom severe abAMR was suspected. Consequently, we identified a patient from 1998 who underwent ABOI-KTx and experienced graft loss due to rejection on POD 51, the clinical course of which is depicted in Supplementary Figure S12. This patient showed a sustained high level of FCM-IgG more than 32 times throughout the course. The FCM-C1q levels gradually increased from POD 27, peaking at 40.5% on POD 50, leading to graft loss on POD 51. There were also patients in whom postoperative FCM-IgG levels were elevated while FCM-C1q levels remained low, leading to either no abAMR or only mild abAMR, as was the case with Cases No. 10 and 17. These patients suggest that a certain level of complement-binding ab-Abs is necessary for abAMR to occur. Furthermore, these patients indicate that elevated FCM-IgG levels after ABOI-KTx do not necessarily lead to abAMR when FCM-C1q values remain low. In support of this hypothesis, a review of historical records revealed a patient from 2002. This patient underwent ABOI-KTx and had consistently high FCM-IgG titers, up to 128 times, while FCM-C1q-positive rates remained no higher than 2.2%, as illustrated in Supplementary Figure S13.

Flow cytometers typically display test values as mean fluorescence intensity (MFI). However, because flow cytometers display fluorescence intensity with varying precision, such as four digits, six digits, or even higher precision, MFI may vary depending on the performance of the analytical instrument. Therefore, we decided to express FCM-C1q method results by the proportion of positive red blood cells to total red blood cells, which is less affected by the performance of the analytical instrument. When evaluating the results of the FCM-C1q test, it is desirable to bring the cut off value as close to 0% as possible. However, based on experiences comparing the results of the FCM-C1q test with the presence or absence of clinical rejection in many cases, a range of 5–10% was set as the indeterminate zone, and a cut off value was set at 10% or higher.

The anti-A/B IgG antibody has subclasses of IgG1, IgG2, IgG3, and IgG4. We have experimentally evaluated these subclasses and found that IgG2 > IgG1 > IgG3 > IgG4 have all been produced in that order in patients with ABOI-KTx before desensitization. By performing desensitization for ABOI-KTx, IgG3 and IgG4 often become negative and IgG2 and IgG1 remain positive while C1q turns negative in most cases. Thus, we hypothesized that although IgG1 has the binding site for C1q, it is not in a state that allows C1q to bind there. In other words, such IgG1 antibodies are those without C1q activity, which do not result in abAMR. When conducting a confirmatory randomized controlled trial to verify the utility of FCM-C1q, it will be necessary to measure the levels of total IgG and each subclass of IgG and verify this hypothesis.

In ABOI-KTx so far, the focus has been only on reducing the titers of anti-A/B IgG antibodies to prevent abAMR. Our newly developed FCM-C1q provides a perspective on what percentage of anti-blood group antibodies possess C1q fixing ability and could potentially cause abAMR. Therefore, to prevent abAMR in ABOI-KTx, it is important not only to reduce the titer of anti-blood group antibodies but also to decrease those antibodies that have C1q activity. Even if the titer of anti-blood group antibodies is high, if the C1q activity is low, the likelihood of moderate or severe abAMR occurring can be considered low. Conversely, even if the anti-blood group antibody titer can be reduced at the time of transplant, if the C1q activity is high, and if the antibody titer rebounds post-surgery with high C1q activity, the possibility of such an abAMR occurring is high. Furthermore, by measuring antibody activity through antibodies’ ability to interact with C1q, regardless of whether these antibodies target HLA or blood group antigens, we can adopt a uniform approach. This perspective allows us to focus primarily on reducing all antibodies that interact with C1q to prevent different types of AMR.

This study has several limitations. There were only limited abAMR samples owing to the rarity of severe abAMR to validate the prognostic efficacy of FCM-C1q. In facilities where the number of ABOI-KTx is low, there is a high likelihood of never encountering such patients. Owing to its retrospective nature, this study solely focused on patients who developed or were suspected of AR including abAMR without fully excluding other causes of renal dysfunction, such as the recurrence of the primary disease. Moreover, there were limited patients in whom renal biopsy could be performed, making it difficult to determine whether abAMR had occurred. One reason is that during the peak of AR, the graft swells due to inflammation and edema, and renal biopsy could injure the capsule. Hence, biopsy is refrained until the graft adheres to the surrounding tissue. However, we assert that the greatest importance of reporting this pilot study lies in widely publicizing this new testing method that is capable of detecting severe abAMR and helping prevent rare but fatal cases of abAMR like HAR. Subclasses of anti-A/B IgG antibody were not measured. Finally, there has not been a consensus on whether HAR could occur in ABOI-KTx, possibly because even if HAR occurs, there are few methods to confirm it. Therefore, we suspect an underreporting of HAR and propose that our novel method may work as a test to determine the presence or to predict the occurrence of moderate to severe abAMR in ABOI-KTx. Future randomized controlled trials are needed to validate the efficacy of the new biomarker FCM-C1q and to standardize this new assay.

## 5. Conclusions

We have developed a novel technique to measure the complement-fixing ability of anti-A/B blood type IgG antibodies for detecting moderate to severe AMR caused by anti-blood type antibodies in ABOI-KTx. The findings suggest that a high positivity rate for the complement-fixing ability of these antibodies along with high FCM-IgG titers could predict moderate to severe abAMR and poor graft survival in ABOI-KTx. Meanwhile, a high FCXM-IgG level does not necessarily indicate a high likelihood of abAMR when FCM-C1q levels are low.

## Figures and Tables

**Table 1:** Background characteristics of Cohort A.

Healthy Subjects (*N* = 44)			Dialysis Patients (*N* = 43)		
Blood type	Sex (M/F)	Age	Blood type	Sex (M/F)	Age
O	9/5	54.5 ± 12.7 (25–70)	O	11/4	54.3 ± 11.3 (34–75)
A	7/8	54.5 ± 13.4 (26–75)	A	13/1	60.8 ± 9.6 (37–70)
B	5/10	54.9 ± 12.5 (38–75)	B	10/4	51.5 ± 11.4 (24–66)

There are no significant differences in the distribution of age or sex across blood types in each group.

**Table 2:** A comparison of patient background characteristics between the rejection group and the non-rejection group in Cohort B.

Variable	Rejection Group (*N* = 16)		Non-Rejection Group (*N* = 46)		*p*-Value
Continuous variables	Average	Range	Average	Range	
Donor age	61.9 ± 10.7	44~79	60.4 ± 8.6	40~78	0.601
Recipient age	48.3 ± 14.9	17~70	49.0 ± 13.7	20~77	0.782
Dialysis period (yr)	6.48 ± 8.69	0.01~23.9	4.39 ± 6.44	0.01~29.6	0.489
HLA A locus AA mismatch	16.6 ± 9.5	0~34	11.7 ± 11.1	0~34	0.171
HLA B locus AA mismatch	12.9 ± 3.9	7~21	10.44 ± 6.7	0~23	0.26
HLA DR locus AA mismatch	10.8 ± 7.4	0~24	11.1 ± 7.8	0~25	0.885
HLA amino acid mismatch	40.1 ± 15.2	10~67	33.2 ± 18.4	0~76	0.133
FCM-IgG before KTx (2^n)	10.0 ± 21.21	1~64	5.48 ± 7.66	1~32	0.647
FCM-C1q before KTx (%)	5.69 ± 16.21	0.4~66	3.59 ± 9.70	0.36~64.9	0.879
POD of FCM test before KTx	−1.56 ± 1.09	−4~0	−1.22 ± 2.11	−14~0	0.072
FCM-IgG after KTx (2^n)	7.81 ± 16.85	1~64	2.67 ± 3.60	1~16	0.52
FCM-C1q after KTx (%)	4.49 ± 10.85	0.45~41.7	0.91 ± 0.53	0.41~3.96	0.748
POD of FCM test after KTx	5.25 ± 2.24	3~12	5.15 ± 2.40	3~18	0.955
Discrete variables	Count	Rate (%)	Count	Rate (%)	
Donor sex (M/F)	5/11	31.3%/68.8%	15/31	32.6%/67.4%	0.92
Recipient gender (M/F)	12/4	75.0%/25.0%	30/16	65.2%/34.8%	0.544
Spousal donor	7	43.8%	25	54.3%	0.465
CDC T-cell	0	0.0%	0	0%	1
CDC B-cell warm	1	6.3%	2	4.3%	1
CDC B-cell cold	1	6.3%	5	10.9%	1
FCXM T-cell	0	0.0%	4	8.7%	0.564
FCXM B-cell	1	6.3%	4	8.7%	1
PRA Class 1	1	6.3%	4	8.7%	1
PRA Class 2	1	6.3%	3	6.5%	1
Preformed DSA	2	12.5%	4	8.7%	0.643
Induction with Basiliximab	16	100.0%	45	97.8%	1
Induction with Rituximab	15	93.8%	44	95.7%	1
Induction with ATG	5	31.3%	5	10.9%	0.0562

AA mismatch, amino acid mismatch; FCM-IgG, flow cytometry method for the IgG test; FCM-C1q, flow cytometry method for the complement C1q test; CDC, complement-dependent cytotoxic crossmatch test; FCXM, flow cytometry lymphocyte crossmatch; PRA, panel-reactive antibody test; DSA, donor-specific antibody; ATG, anti-thymocyte globulin. Preformed DSA refers to DSA that existed before transplantation.

## Data Availability

Data is contained within the Supplementary Materials.

## Supplementary Materials

The following supporting materials can be downloaded at: https://www.mdpi.com/article/10.3390/antib13030062/s1, Table S1: Clinical courses of 62 study participants; Table S2: the immunological test results and clinical courses of all cases; Figure S1: Procedures for dilutions in FCM-IgG; Figure S2: Method for counting positive cells in FCM-C1q; Figure S3: Immunological regimen for ABOI-KTx; Figure S4: Macroscopic view of removed kidney of Case No. 1; Figure S5: Microscopic view of removed kidney of Case No. 1; Figure S6: Clinical course of Case No. 1; Figure S7: Clinical course of Case No. 2; Figure S8: Clinical course of Case No. 3; Figure S9: Clinical course of Case No. 10; Figure S10: Clinical course of Case No. 17; Figure S11: Clinical course of Case No. 18; Figure S12: Clinical course of historical case with AMR; Figure S13: Clinical course of historical case without AMR.

## Abbreviations

ABOI-KTx, ABO blood type-incompatible kidney transplantation; Ars, acute rejections; ab-Abs, anti-blood type antibodies; ah-Abs, anti-HLA antibodies; AMR, antibody-mediated rejection; abAMR, anti-blood type antibody-mediated rejection; ATG, anti-thymoglobulin; aHUS, atypical hemolytic uremic syndrome; BXM, basiliximab; RXM, chimeric anti-CD20 monoclonal antibody rituximab; DTT, dithiothreitol; DSAs, donor-specific anti-HLA-IgG antibodies; DFPP, double-filtration plasmapheresis; FCM-C1q, flow cytometry method for the complement C1q test; FCM-IgG, flow cytometry method for the IgG test; FITC, fluorescein isothiocyanate; HBV, hepatitis B virus; hcc-C1q, human complement component C1q; HAR, hyperacute antibody-mediated rejection; MP, methylprednisolone; MMF, mycophenolate mofetil; NRG, non-rejection group; PPx, plasmapheresis; POD, postoperative day; RBC-S, red blood cell suspension; RG, rejection group; s-Cr, serum creatinine; sAR, suspected acute rejection; Tac, tacrolimus; TMR, T-cell-mediated rejection; TMA, thrombotic microangiopathy.
