# Supplementary figures and images for: Novel Flow Cytometry Method Detecting Complement C1q Bound to Blood Type A/B IgG Antibody for Preventing Severe Antibody-Mediated Rejection in ABO-Incompatible Kidney Transplantation
Source: Antibodies (Basel). 2024 Aug 1;13(3):62. doi: 10.3390/antib13030062 (PMC11348181; doi:10.3390/antib13030062)

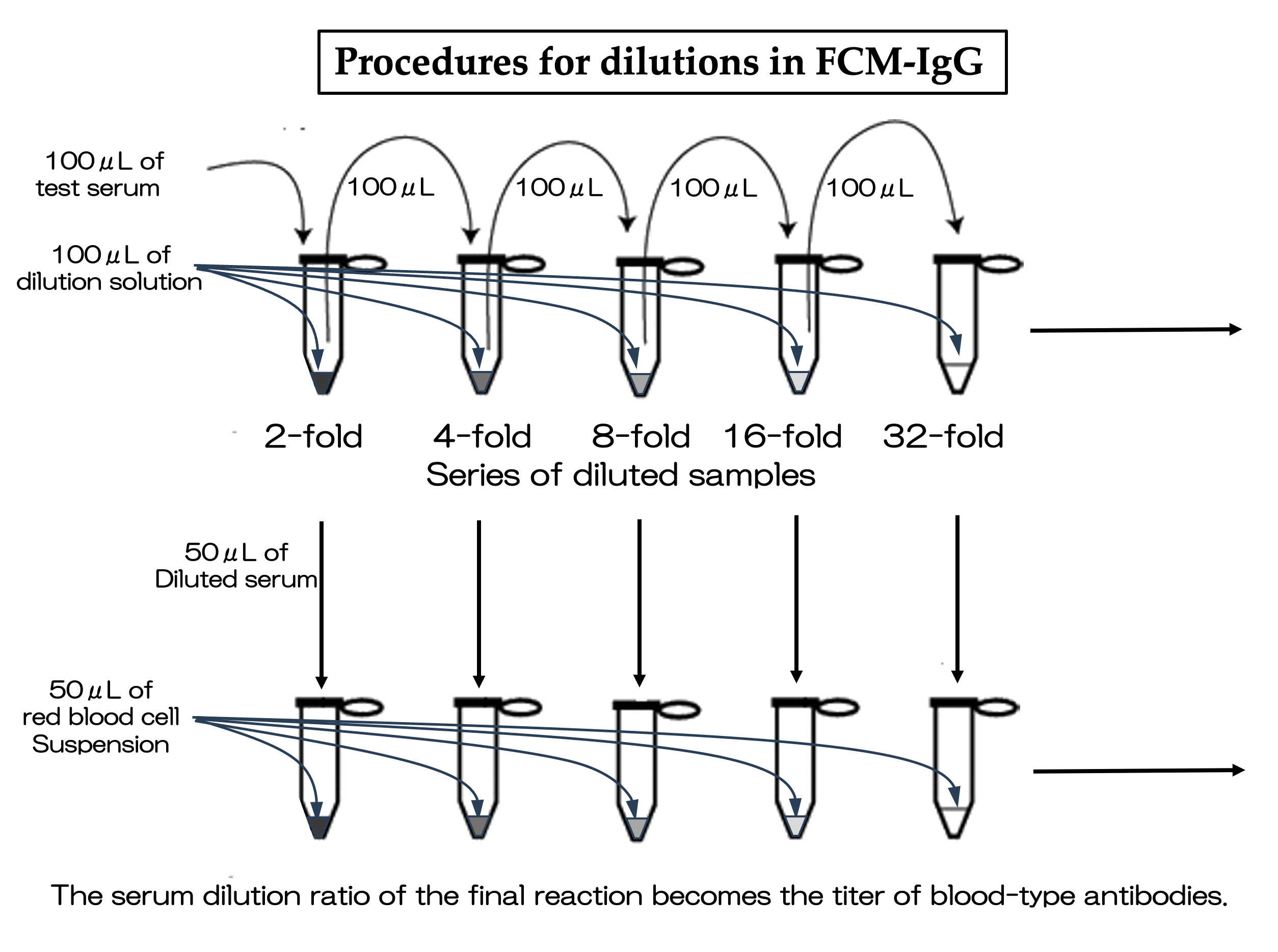

Supplement: Supplementary file 1 [file antibodies-13-00062-s001.zip › SupplementaryFigureS1.png]

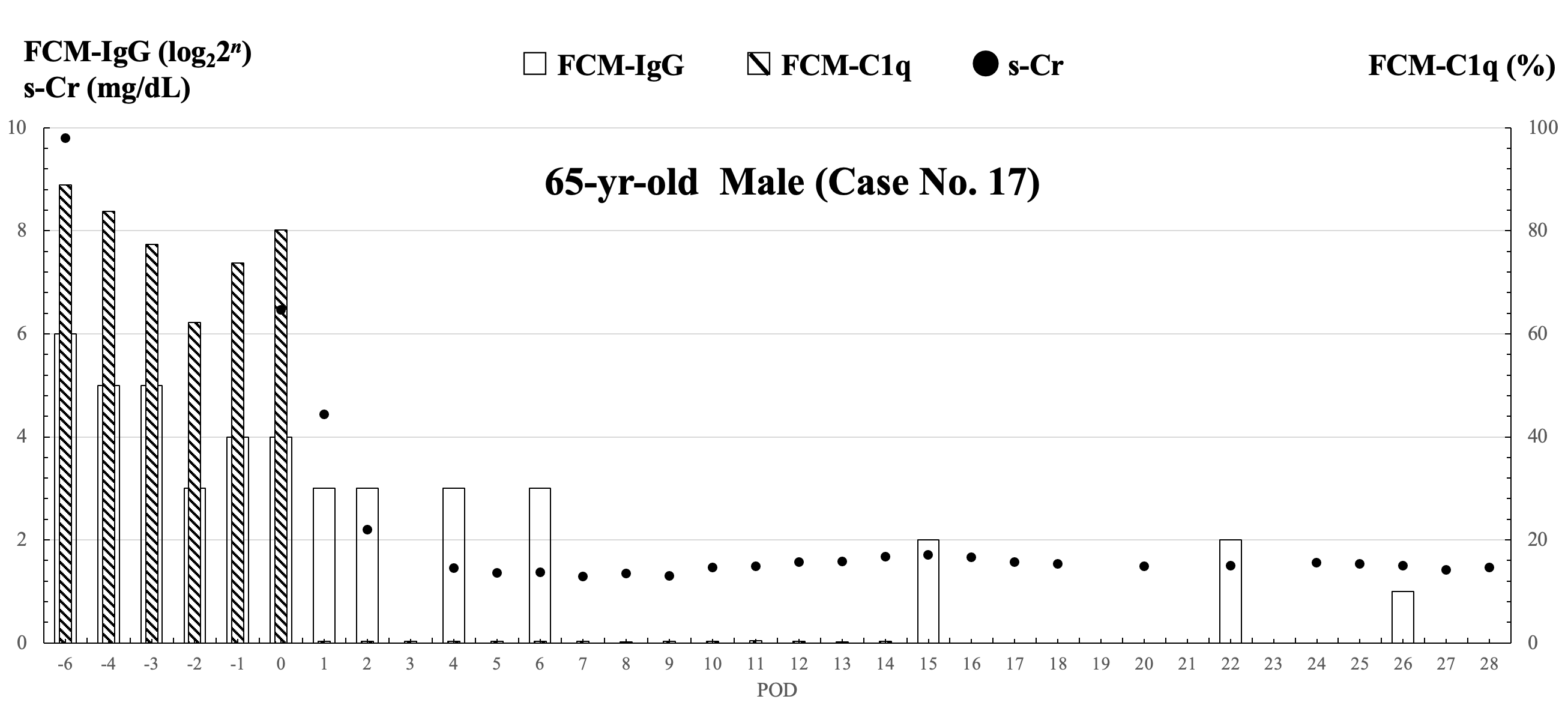

Supplement: Supplementary file 1 [file antibodies-13-00062-s001.zip › SupplementaryFigureS10shifted.png]

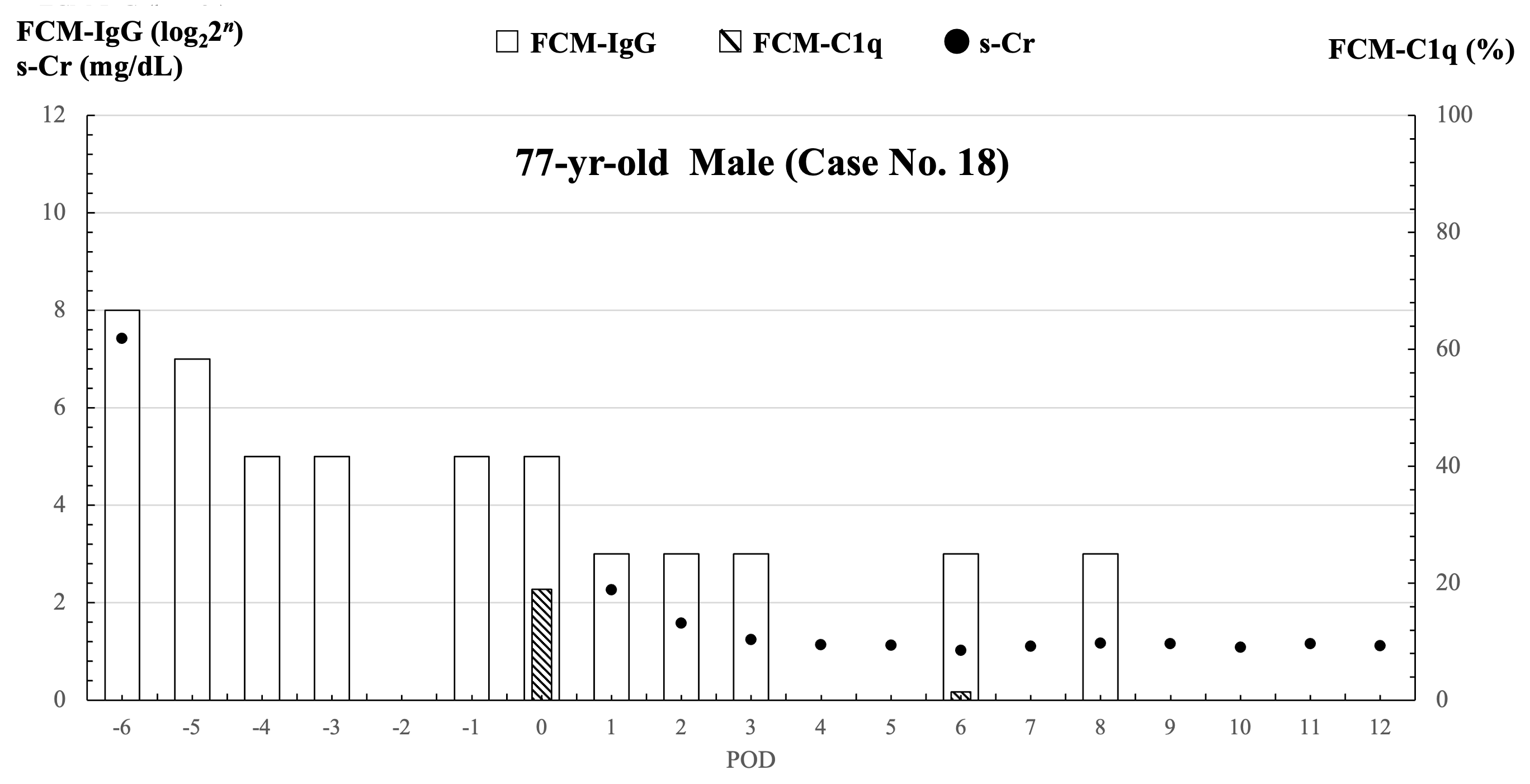

Supplement: Supplementary file 1 [file antibodies-13-00062-s001.zip › SupplementaryFigureS11shifted.png]

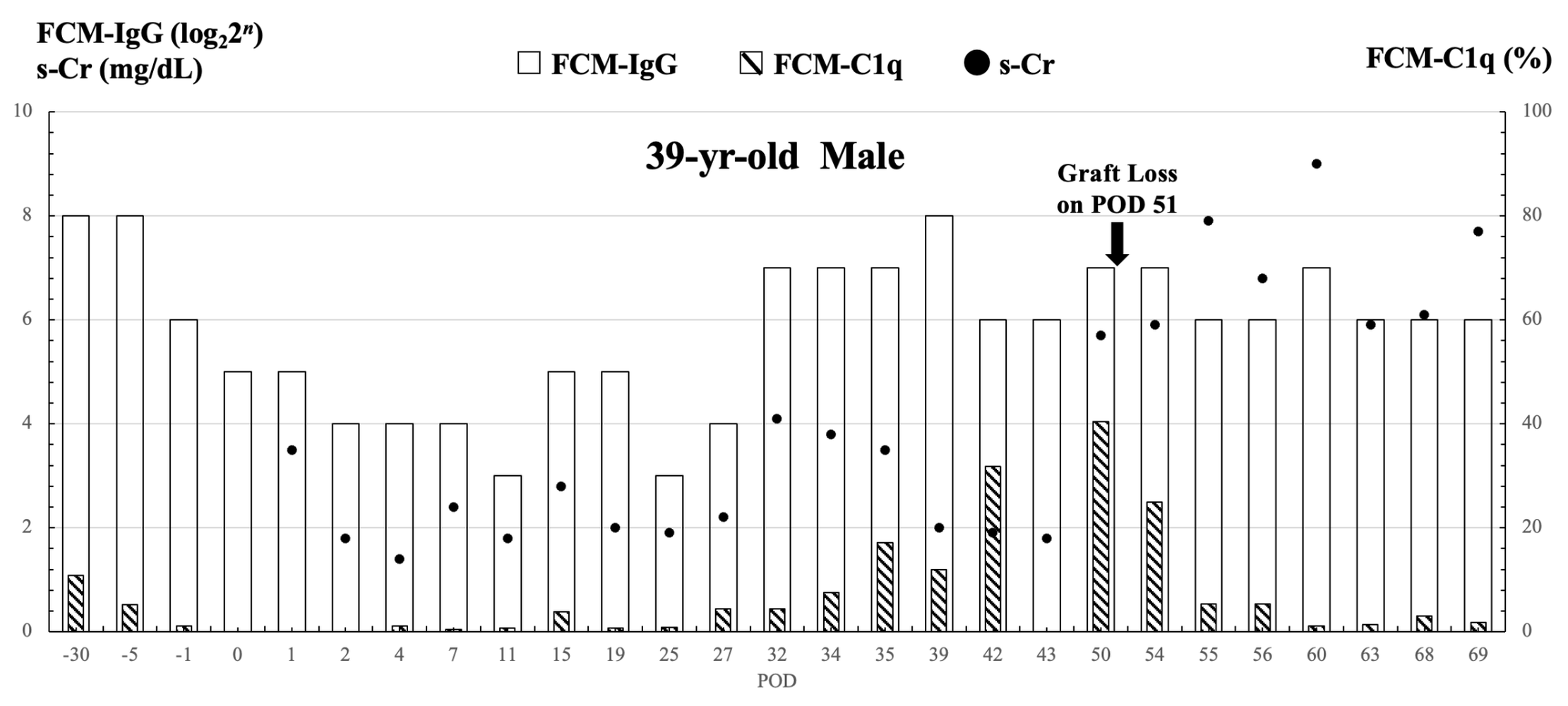

Supplement: Supplementary file 1 [file antibodies-13-00062-s001.zip › SupplementaryFigureS12shifted.png]

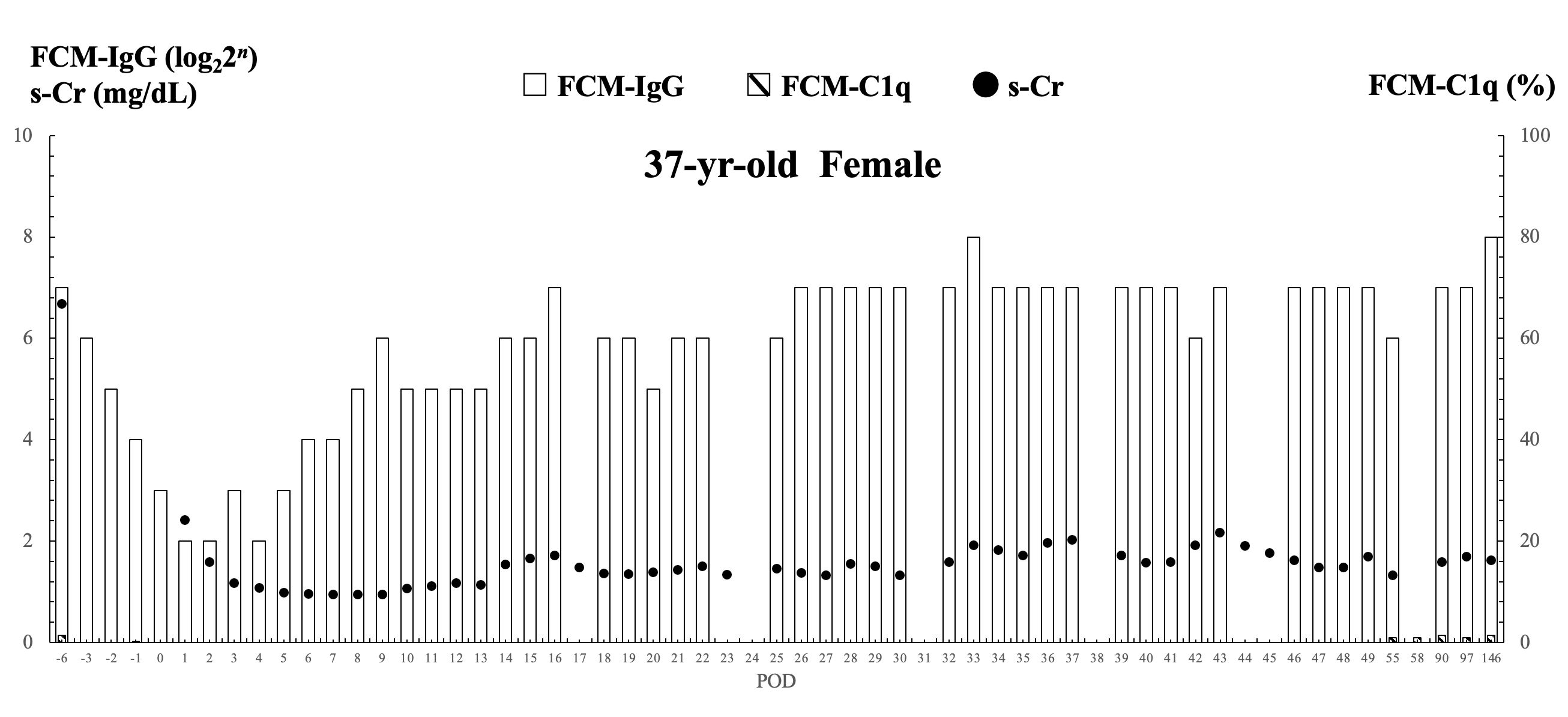

Supplement: Supplementary file 1 [file antibodies-13-00062-s001.zip › SupplementaryFigureS13shifted.png]

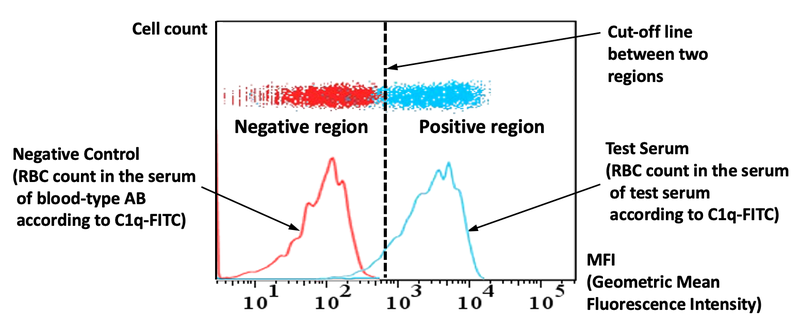

Supplement: Supplementary file 1 [file antibodies-13-00062-s001.zip › SupplementaryFigureS2new.png]

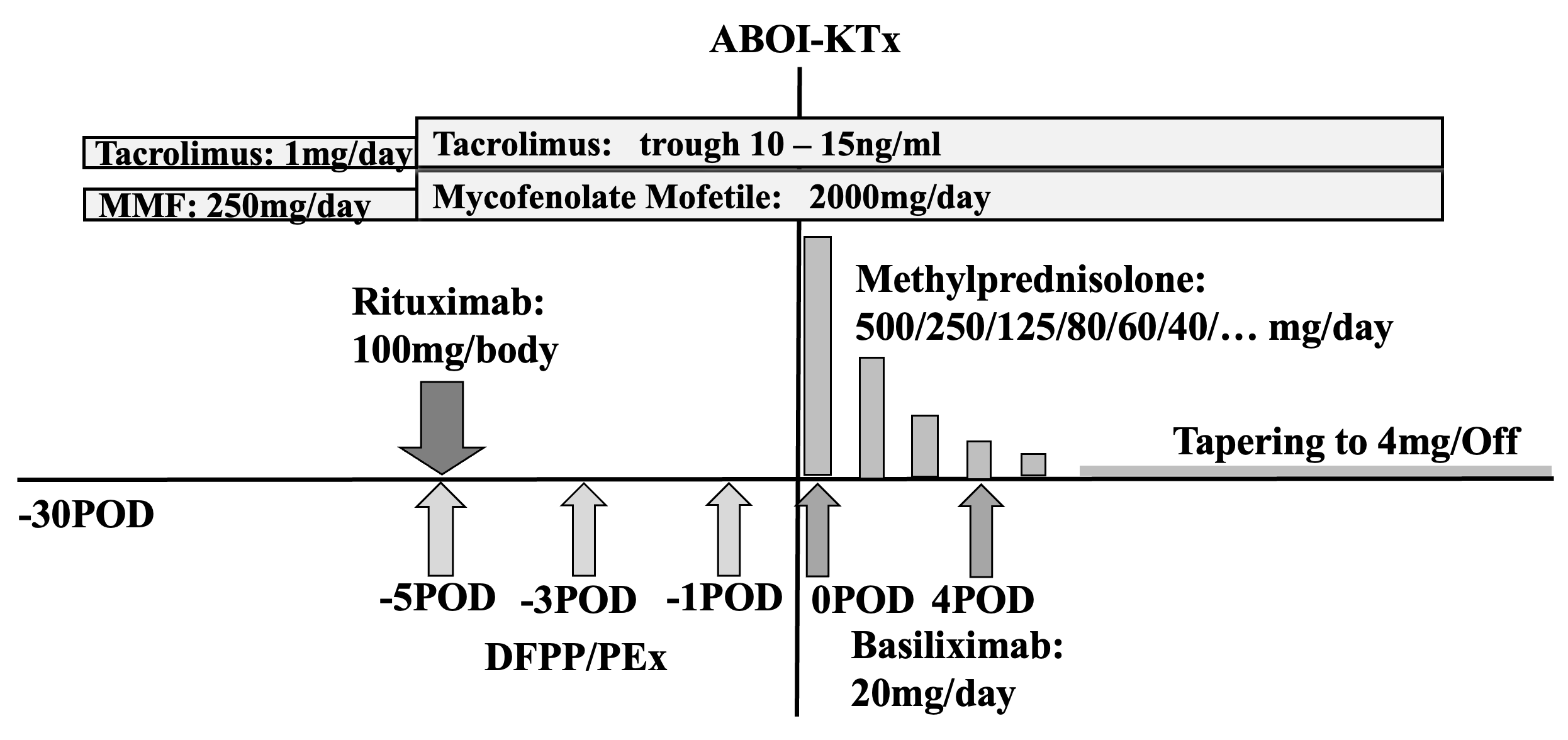

Supplement: Supplementary file 1 [file antibodies-13-00062-s001.zip › SupplementaryFigureS3shifted.png]

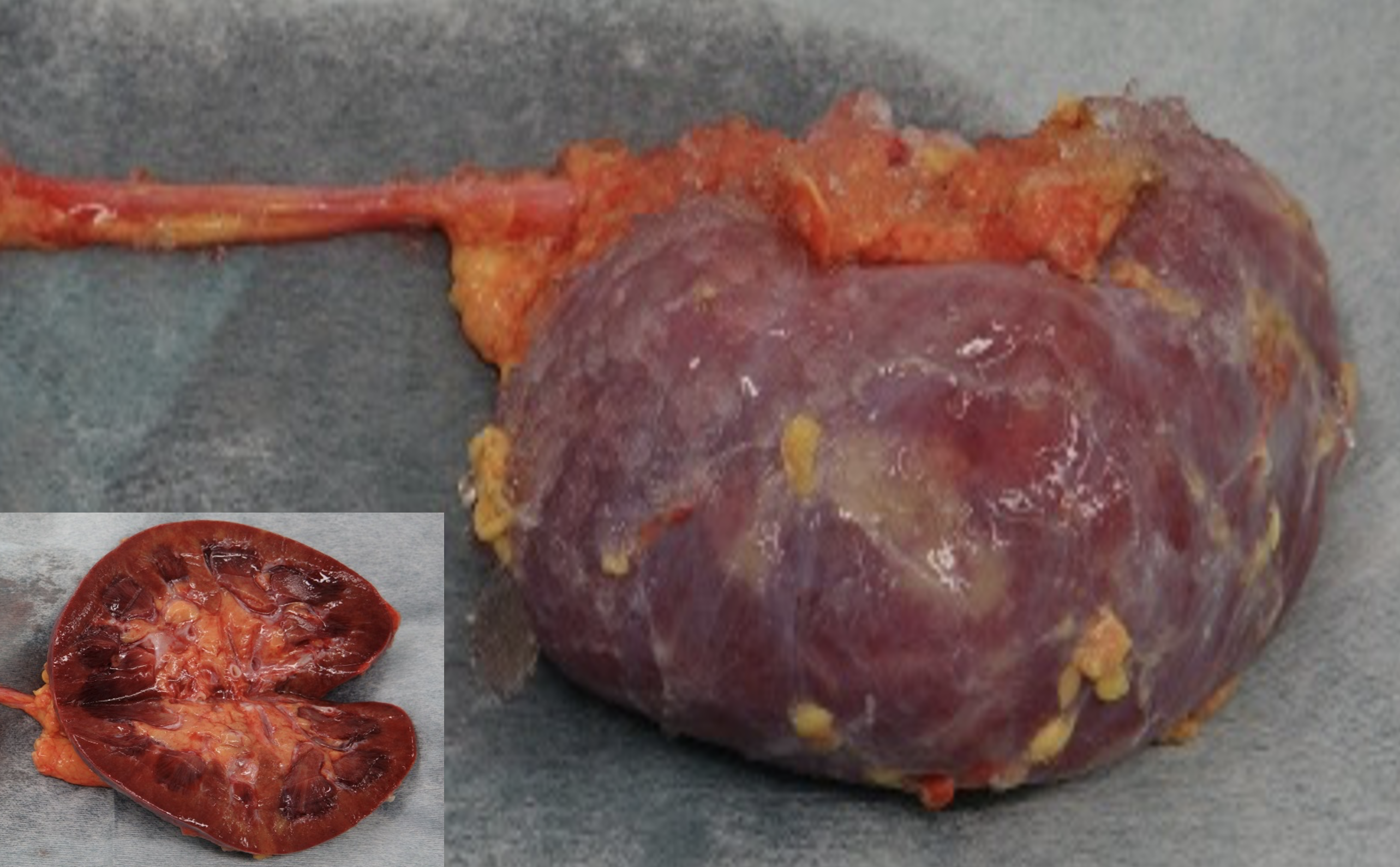

Supplement: Supplementary file 1 [file antibodies-13-00062-s001.zip › SupplementaryFigureS4shifted.png]

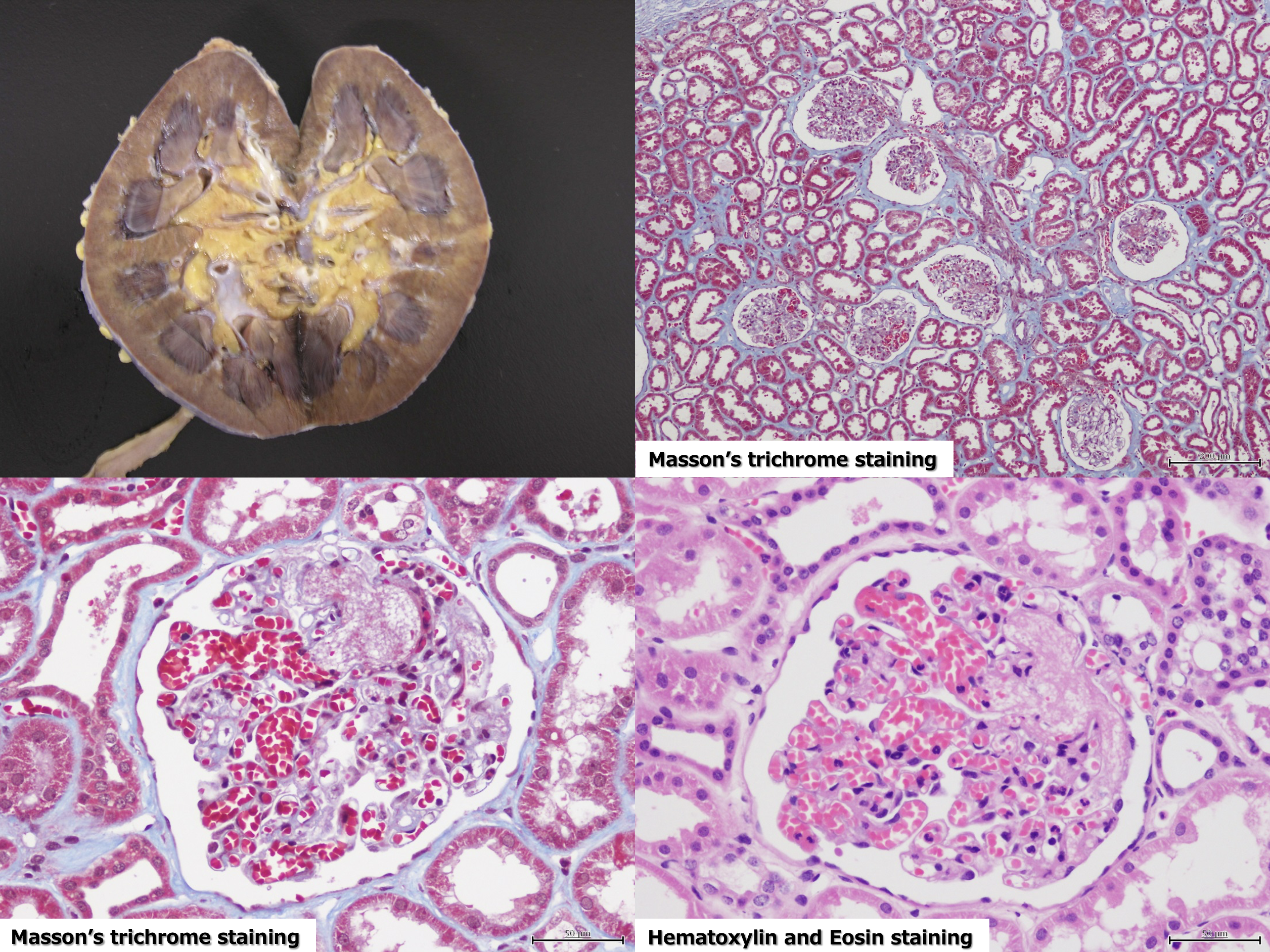

Supplement: Supplementary file 1 [file antibodies-13-00062-s001.zip › SupplementaryFigureS5shifted.png]

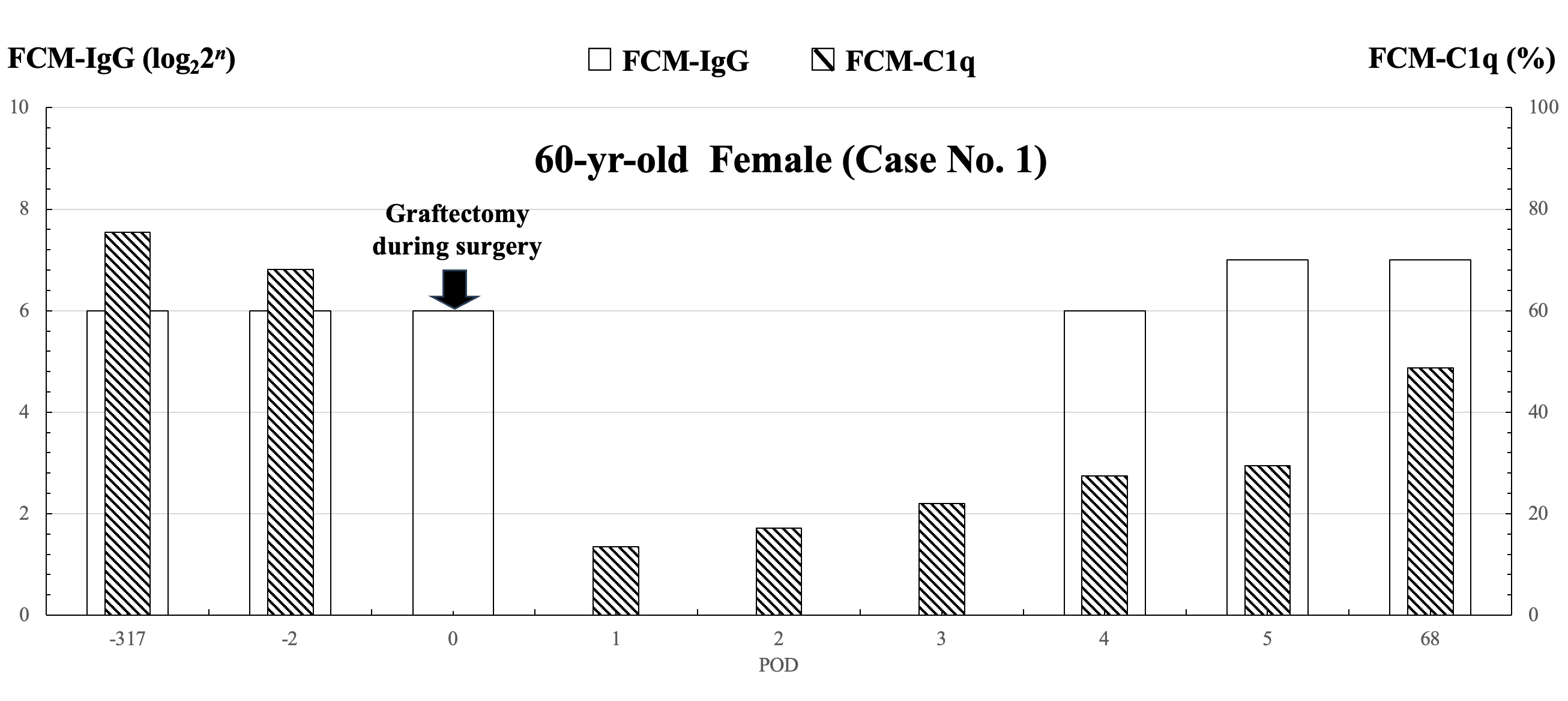

Supplement: Supplementary file 1 [file antibodies-13-00062-s001.zip › SupplementaryFigureS6shifted.png]

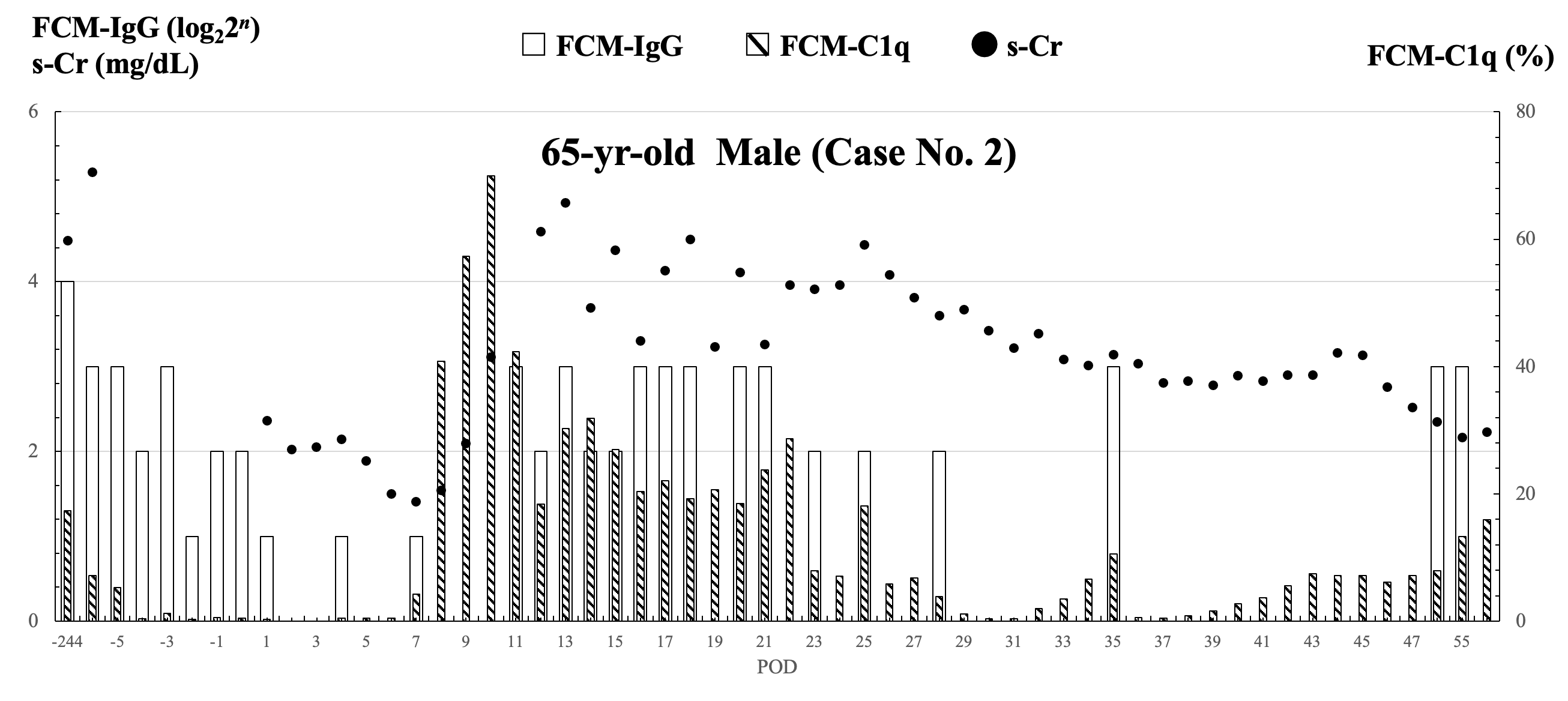

Supplement: Supplementary file 1 [file antibodies-13-00062-s001.zip › SupplementaryFigureS7shifted.png]

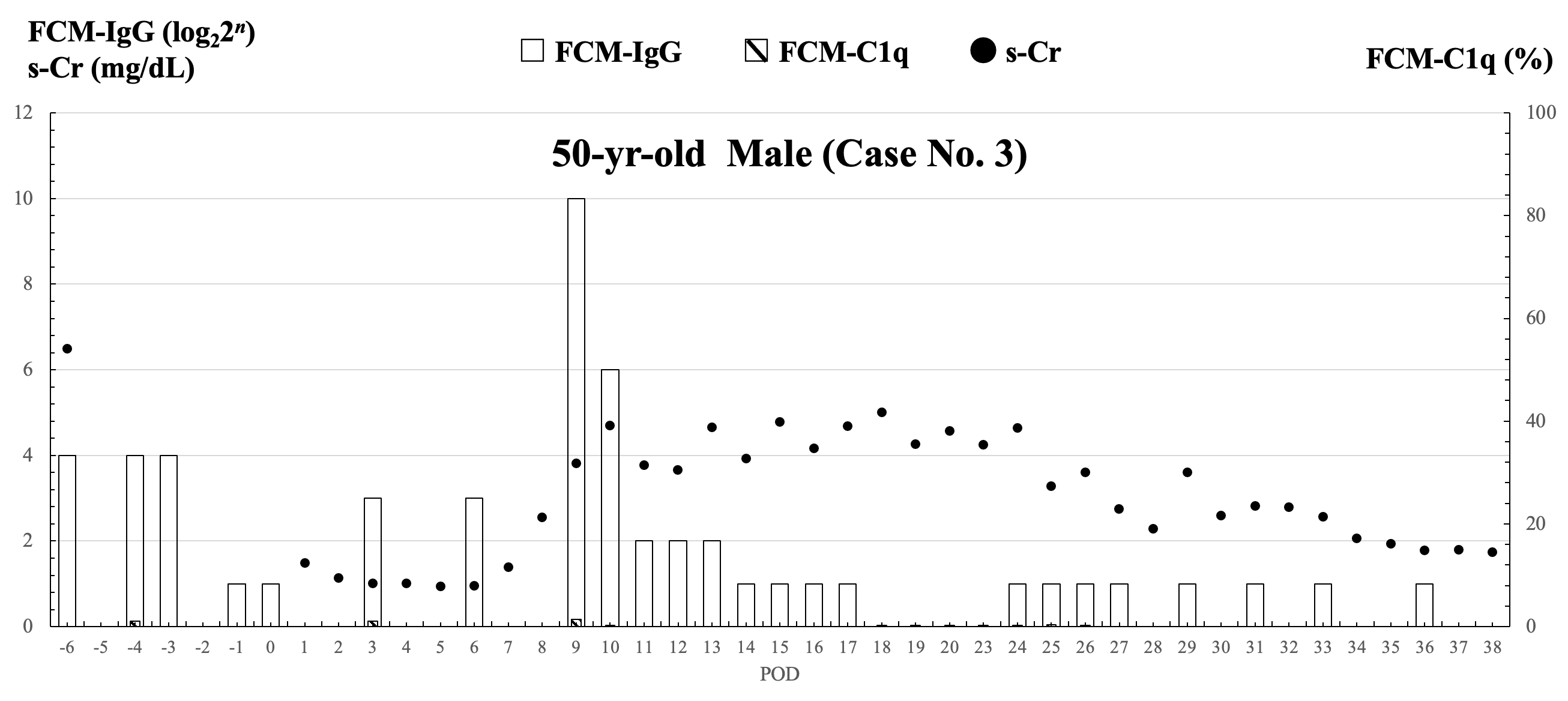

Supplement: Supplementary file 1 [file antibodies-13-00062-s001.zip › SupplementaryFigureS8shifted.png]

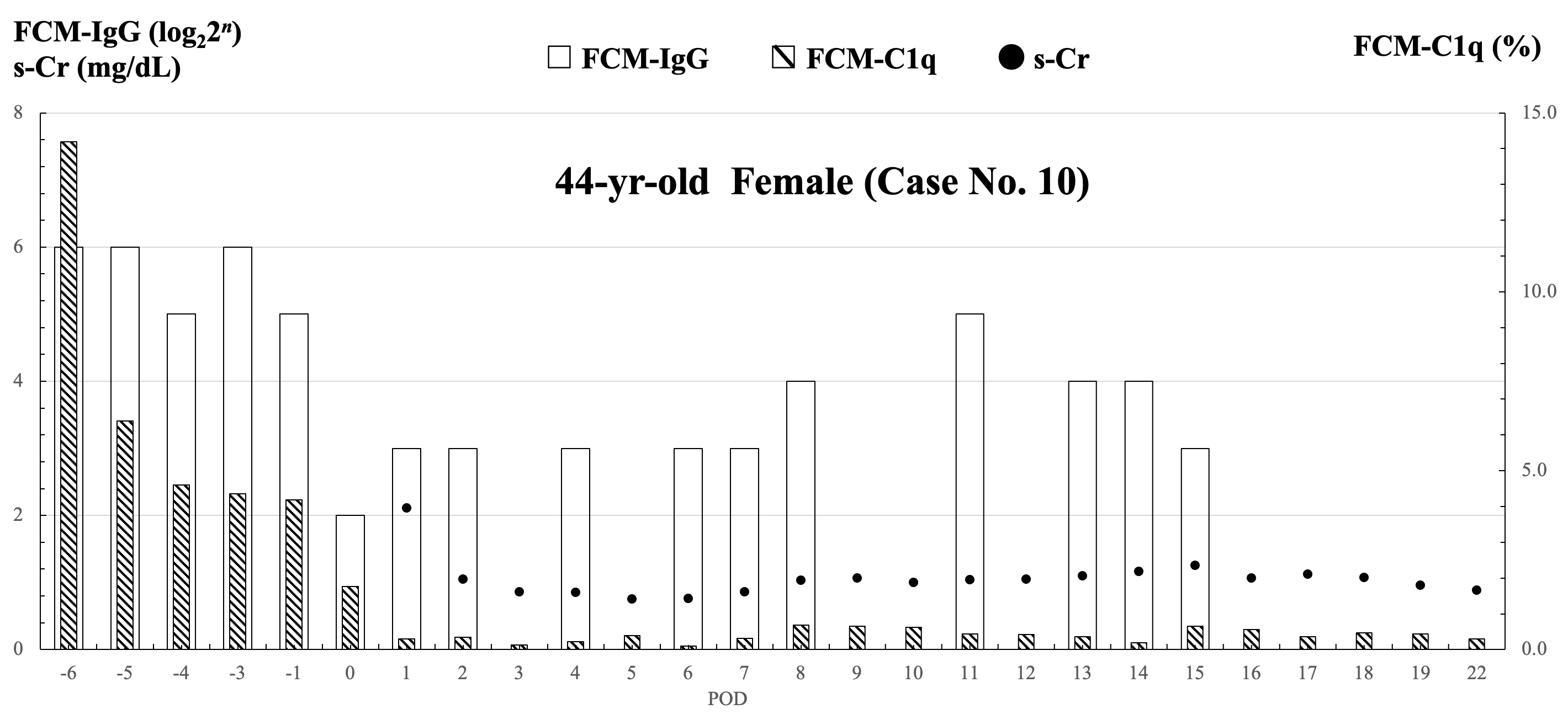

Supplement: Supplementary file 1 [file antibodies-13-00062-s001.zip › SupplementaryFigureS9shifted.png]
